# Addition of P3HT-grafted Silica nanoparticles improves bulk-heterojunction morphology in P3HT-PCBM blends

**DOI:** 10.1038/srep33219

**Published:** 2016-09-15

**Authors:** Mohit Garg, Venkat Padmanabhan

**Affiliations:** 1 Department of Chemical Engineering, Indian Institute of Technology, Kharagpur 721302, India

## Abstract

We present molecular dynamics simulations of a ternary blend of P3HT, PCBM and P3HT-grafted silica nanoparticles (SiNP) for applications in polymer-based solar cells. Using coarse-grained models, we study the effect of SiNP on the spatial arrangement of PCBM in P3HT. Our results suggest that addition of SiNP not only alters the morphology of PCBM clusters but also improves the crystallinity of P3HT. We exploit the property of grafted SiNP to self-assemble into a variety of anisotropic structures and the tendency of PCBM to preferentially adhere to SiNP surface, due to favorable interactions, to achieve morphologies with desirable characteristics for the active layer, including domain size, crystallinity of P3HT, and elimination of isolated islands of PCBM. As the concentration of SiNP increases, the number of isolated PCBM molecules decreases, which in turn improves the crystallinity of P3HT domains. We also observe that by tuning the grafting parameters of SiNP, it is possible to achieve structures ranging from cylindrical to sheets to highly interconnected network of strings. The changes brought about by addition of SiNP shows a promising potential to improve the performance of these materials when used as active layers in organic photovoltaics.

Organic photovoltaics (OPV) have gained significant interest in the past few decades as a potential alternate source of energy due to use of low cost materials, inexpensive fabrication techniques and prospects of flexible solar panels^1^,^2^,^3^,^4^,^5^. However, the last few years have witnessed a substantial decrease in interest due to the apparent stagnation of the efficiency of these cells. The photoactive layer of an OPV device typically consists of a bulk-heterojunction (BHJ) that comprises blends of conjugated polymers as electron donors and functionalized fullerenes as electron acceptors. Currently, systems consisting of regio-regular poly(3-hexylthiophene) (P3HT) as electron donor and (6,6)-phenyl-C61-butyric acid methyl ester (PCBM) as electron acceptor are found to be most promising for BHJs, both in terms of efficiency and long-term stability^6^,^7^. The success of polymer-based solar cells relies partly on the formation of phase separated micro-structures with high charge carrier mobility^8^,^9^,^10^. Although, the efficiency of these cells has increased from 1% in a conventional single layer solar cell to about 11% in BHJ cells^11^,^12^, it is still significantly lower than that of their inorganic counterparts and insufficient to attain an economic viability in the market.

In a typical OPV device, incident photons are absorbed by the electron donor in the active layer and coloumbically-bound electron-hole pairs, known as excitons, are created^13^. These excitons are then split into free carriers at the interface between donor and acceptor materials, which are then transported to the corresponding electrodes resulting in a photocurrent. Apart from the absorption spectrum of the donor material, there are two other important factors that limit the efficiency of these cells. The length scale for exciton diffusion without recombination has been found to be ~10 nm^14^,^15^. Hence, excitons must find a donor-acceptor interface within this distance from its point of origin to minimize the losses due to recombination. Secondly, a continuous percolated network of donor and acceptor materials is critical for successfully transferring the dissociated carriers to their respective electrodes^16^,^17^,^18^. Based on these factors, it has been suggested that a ‘comb-shaped’ structure, with ~10 nm gap between each tooth, of the acceptor material in the active layer is optimum for maximum possible efficiency of OPVs^19^.

Several theoretical^20^,^21^,^22^,^23^,^24^,^25^ and experimental^26^,^27^,^28^,^29^,^30^,^31^,^32^ studies exist that focus on improving the spatial arrangement of polymers and nanoparticles in bulk^21^,^24^,^31^ and in films^26^,^32^,^33^,^34^. The morphology of constituents in the photoactive layer can be strongly affected by various factors and processing conditions; such as donor-acceptor composition and molecular weight^35^,^36^,^37^,^38^, choice of solvent^39^, rate of solvent evaporation^40^, processing temperature^41^, use of chemical additives^42^, etc. It has been shown that thermal annealing of the photoactive layer results in a higher degree of mesoscopic order and crystallinity of the two domains that can significantly improve the device performance^43^. During the annealing process, a competition between P3HT crystallization and PCBM diffusion controls the phase separation of the two constituents in the blend. It has also been established that a weight ratio of 1:1 is optimum for achieving the desired morphology of constituents within the BHJ^38^. Recent studies have shown that addition of a third component to the donor-acceptor blend shows promising evidence of improvement in the efficiency of the OPV device^44^. Adding an inert polymer like polystyrene to the P3HT-PCBM blend results in the formation of highly ordered columnar structures due to lateral phase separation in thin films^32^. This structure provides high interfacial area for charge transport but lowers the crystallinity of PCBM. It has also been reported that addition of syndiotactic polystyrene can further enhance the efficiency by increasing the crystallization of P3HT^45^. BHJs prepared by grafting P3HT on to the surface of PCBM have shown to improve the thermal stability of the system and obtain domain sizes of around 5 nm. However, chemical modification of the PCBM surface during grafting reduces its optical properties and the small domain size affects the crystallinity of P3HT thereby reducing the efficiency^46^,^47^. Block copolymers consisting of P3HT along with other insulating polymers have shown to increase the thermal stability, crystallinity and formation of appropriately sized nano domains but the efficiencies reported for such BHJs are relatively low due to the presence of insulating polymers^48^,^49^,^50^. A ternary blend of poly-3-oxothieno(3,4-d) isothiazole-1,1-dioxide/benzodithiophene (PID2), polythieno[3,4-b]-thiophene/benzodithiophene (PTB7) and (6,6)-phenyl C71 butyric acid methyl ester (PC_71_BM) has shown to have a higher light absorption due to the conducting nature of both polymers but no significant enhancement in the efficiency was found due to the low hole mobility of PTB7 that also limits the thickness of the active layer to below ~200 nm^51^,^52^. Similarly, metal nanoparticles like gold, silver, and copper, when added to the transparent layer above the active layer showed an increase in the light absorption ability of the active layer^53^,^54^ and copper phthalocyanine (CuPc) nanocrystals, when added to the donor-acceptor blends, act as nucleation sites for crystallization^55^. However, no clear evidence of morphology enhancement was found with CuPc in the blend. In a more recent study, Shen *et al*.^56^ added a single large silica nanoparticle (of size ~133 nm) to the BHJ and found that the morphological changes introduced by the high surface energy of silica was responsible for increasing the efficiency up to 10–20%.

It is clear that a precise control over the spatial arrangement of constituents in the active layer is crucial for improving the efficiency of polymer-based solar cells and an extensive amount of work exists that deals with various strategies to improve the morphology. However in most of the work cited above, especially where a third component is added to the existing P3HT-PCBM blend, although certain advantages are observed, the efficiency of such cells show no significant improvement. This is because the efficiency of polymer-based solar cells depends on various factors and achieving a thorough control over all of them is extremely challenging. For instance, when the third component is a conducting polymer, the light absorption of the active layer shows an increase but the transport of holes to the anode is limited by the lower conductivity of the added component. On the other hand, when an insulating polymer is added, the structural changes observed are promising and closer to the ideal morphology suggested for maximum possible efficiency, but the presence of insulating polymer not only interferes with the charge transfer dynamics of the donor and acceptor materials but also deteriorates the crystallinity of both domains. Hence, when a third component is added to the donor-acceptor blend, it is necessary that the added component offers all the advantages but does not hinder the formation, dissociation, or transport of the charge carriers through the corresponding domains. In this work, we present molecular dynamics (MD) simulations of a ternary blend of P3HT, PCBM and P3HT-grafted silica nanoparticles (SiNP). Here, although SiNPs are insulators, we observe that the right parameters of the grafted chains (including graft density and molecular weight) result in the formation of percolated networks of silica particles and due to their higher surface energy and size, PCBM molecules migrate to the P3HT-SiNP interface resulting in percolated networks of PCBM domains that are essential for smooth transport of electrons to the cathode. As SiNPs are completely covered with PCBM, their presence does not affect the exciton dissociation that only occurs at the P3HT-PCBM interface. In addition to improving the overall structure of PCBM domain, we also note that addition of SiNP reduces the number of isolated PCBM particles within the blend and improves the crystallinity of P3HT domains.

## Methods

The models used for P3HT and PCBM have been adapted from the work of Lee *et al*.^21^. P3HT is modeled as linear chains where each interaction center is represented as one single spherical bead. The force field parameters for bonded interaction include bonds, angles, and dihedrals, which correspond to the hindered rotation chain model^57^. The bonds and angles are governed by harmonic potential and dihedrals by OPLS (optimized potential for liquid simulation). In our simulations, we use chains with 40 beads/chain to maintain a polymer-like behavior of the P3HT chains. The force field parameters for bonded interactions are summarized in Table 1. The values of various parameters in the model equations, derived from systematic coarse graining of the atomistic model for P3HT, provide an accurate representation of the regio-regular P3HT with a persistence length of around 37 Å^21^, which is quite similar to the experimental value^58^. PCBM molecules are modeled as spherical beads, which is also obtained from the coarse graining scheme employed by Lee and co workers^21^. Although it is a lower resolution model, it maintains the structure optimality of PCBM fairly well and also allows us to evolve systems, with experimentally relevant length scales, for longer times.

Silica nanoparticles (SiNP) are modeled following a multiscale computational approach, developed by Lee and Hua^59^, for calculating the non-bonded interaction potential for Si_6_O_12_ by coarse graining it to a single bead. In our simulations, we have considered silica nanoparticles which consist of a number of Si_6_O_12_ beads that are held together by harmonic bonds. To construct an SiNP, we first place the Si_6_O_12_ coarse grained beads in an FCC lattice and then carve a sphere of desired size. The diameter of SiNP, used in this study, is 4.7 nm, which consists of 321 Si_6_O_12_ beads. Grafted SiNP is then constructed by grafting P3HT chains on Si_6_O_12_ beads that are on the surface of SiNP. In this study, we consider four grafting densities (Σ_*g*_ = 0.1, 0.2, 0.27, and 0.35 chains/nm^2^) and four graft lengths (*M*_*g*_ = 10, 20, 30, 40 beads/chain) to study the effect of grafting parameters on the self-assembly. The nonbonded interactions for all components in the system are represented by classical Lennard Jones (LJ) 12–6 potential with the Fender-Halsey mixing rule^60^ applied to inter-species interactions. We confirm that the mixing rule applies satisfactorily to Si_6_O_12_ - P3HT and Si_6_O_12_ - PCBM non-bonded interactions by comparing the partial pair correlation functions obtained from our coarse-grained model to a fully atomistic simulation at similar conditions as shown in Fig. S1. The inter and intra particle nonbonded interaction parameters are listed in Table 2.

All simulations are carried out using the LAMMPS molecular dynamics package developed at Sandia National Laboratory^61^. We prepare our systems for studying the morphology of the ternary blend in a two-step process. In order to minimize the effect of initial configuration on the equilibrated structures, we first generate our systems by randomly placing the appropriate amounts of P3HT, PCBM, and P3HT-grafted SiNP in a three dimensional periodic cubic box of size 80 nm such that the density of our initial system is 0.16 g/cm^3^. This is significantly lower than the experimental density of the active layer used in the polymer-based solar cells^62^. Although, it has been reported that a P3HT:PCBM weight ratio of 1:1 is highly desirable for a good phase separation and overall morphology of the constituents within the active layer^63^, in this study we use a P3HT:PCBM weight ratio of 4:1. This is justifiable because, the focus of this work is to emphasize the addition of P3HT-grafted SiNP to the P3HT-PCBM blends to achieve a greater control over the morphology and improve the overall performance of the cell. We used three different weight fractions of SiNP (0.07, 0.1, and 0.13) in our simulations to study the effect of SiNP concentration on the morphology. Once the first stage is complete, the system energy is minimized in an NVE ensemble using Langevin thermostat with a coupling constant of 1 ps. This ensures that all systems are thoroughly random and have no influence of the initial state. The low density of the system facilitates faster equilibration and randomization of the systems. In the next stage of equilibration, we switch to the isothermal-isobaric (NPT) ensemble and the systems are equilibrated for 2 *μ*s to obtain a thermodynamically stable state at a temperature T = 150 °C and pressure P = 1 atm and the final stable density of system is achieved using the Nose-Hoover thermostat and barostat with coupling constants of 0.1 ps and 1 ps, respectively. The time step used in all simulations is 10 fs. The data for analysis are then collected from subsequent production runs of 10 ns in an NPT ensemble with an output frequency of 100 ps.

## Results and Discussion

**Figure 1:**
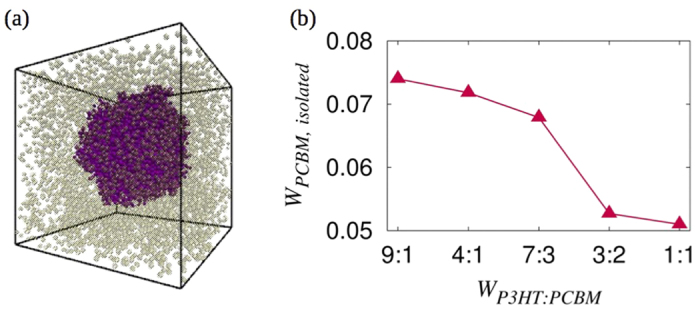
(**a**) Spherical aggregate of PCBM in P3HT. The transparent spheres represent the isolated PCBM molecules. (**b**) Weight fraction of isolated PCBM as a function of P3HT:PCBM weight ratio.

The efficiency of polymer-based solar cells depends greatly on the morphology of acceptor molecules in the polymer matrix. Based on the exciton diffusion length, a comb-shaped structure of PCBM is ideal for maximizing the dissociation of electrons and holes. However in reality, the spatial arrangement of PCBM in P3HT is far from the ideal morphology as shown in Fig. 1a. Although several strategies, such as, choice of solvent, annealing, etc., have improved the formation of bulk heterojunction, the weak phase separation of P3HT-PCBM blends not only creates islands of spherical PCBM clusters (represented by pink spheres) that are not useful in successfully converting light to electricity, but also the dispersion of several PCBM molecules (represented by transparent grey spheres) affects the crystallinity of polymer domains, which in turn hinders the formation and transport of excitons. Clusters of this type are isolated and a majority of them form away from the cathode causing an electron, dissociated at the donor-acceptor interface, to remain until it is eventually combined with a hole. This is one of the major reasons for recombination losses second only to the non-availability of a donor-acceptor interface within the exciton diffusion length. In Fig. 1b we show the variation of dispersed PCBM molecules as a function of P3HT:PCBM weight ratio. It is clear that the number of PCBM molecules dispersed in the matrix decreases with increase in the concentration of PCBM and is in excellent agreement with earlier studies^64^.

### Effect of P3HT-grafted SiNP on PCBM morphology

**Figure 2:**
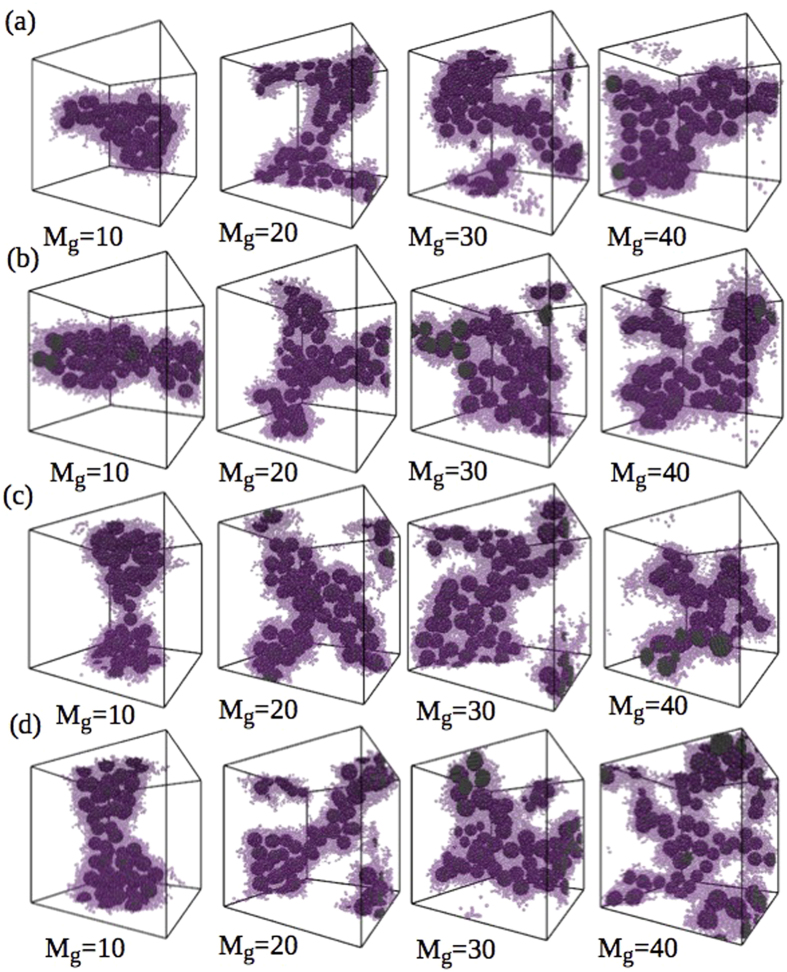
Equilibrated structures of SiNP and PCBM in P3HT. The transparent pink spheres represent PCBM and the opaque gray particles represent SiNP in systems with *W_SiNP_* = 0.1 and Σ*_g_* = a) 0.1, b) 0.2, c) 0.27, and d) 0.35. The graft length (*M_g_*) varies along the columns as indicated in the figure. The isolated PCBM molecules and P3HT are not shown for clarity.

In order to improve not only the degree of phase separation in P3HT-PCBM blends but also the overall morphology of PCBM clusters in the matrix, we added P3HT-grafted silica nanoparticles (SiNP) to the existing blend. The choice of SiNP as a third component in the active layer is based on its high surface energy and ease of grafting the SiNP surface with a variety of polymers experimentally. Earlier experiments have shown that gold nanoparticles preferentially adhere to functionalized silica nanoparticles giving a greater control over their spatial arrangement^65^. It has been shown that SiNPs with no grafting predominantly form spherical aggregates while those that are grafted with polymers self-assemble into a variety of anisotropic structures when the graft parameters, including the grafting density and molecular weight, are carefully tuned^66^,^67^,^68^. Similar works on PGMA-grafted TiO_2_^69^ and polymer-grafted gold nanoparticles^70^ have shown the formation of string-like tubular structures for intermediate values of grafting density and molecular weight. In context of the present study, as the prime objective is to modify the morphology of PCBM in P3HT that can improve the overall conversion efficiency of the cell, grafted SiNP delivers useful properties that gives us a greater control over the morphology. In addition, it also offers the advantage of better control without the requirement for chemical modification of the species (P3HT and PCBM) that are involved in the proper functioning of the active layer. Figure 2 shows the snapshots of equilibrated systems with a weight fraction of P3HT-grafted SiNP, *W*_*SiNP*_ = 0.1 for various graft densities and lengths. The snapshots for *W*_*SiNP*_ = 0.07 and 0.13 (Figs S1 and S2, respectively) are presented in the Supplementary Information (SI). In all the snapshots, we have removed the P3HT chains and isolated PCBM molecules for better visualization of the effect of SiNP on the formation of PCBM clusters. We observe that in all cases, addition of SiNP drastically improves the morphology and results in the formation of well-defined clusters. However, the quality and shape of the clusters are greatly dependent on the concentration and grafting parameters of SiNP.

**Figure 3:**
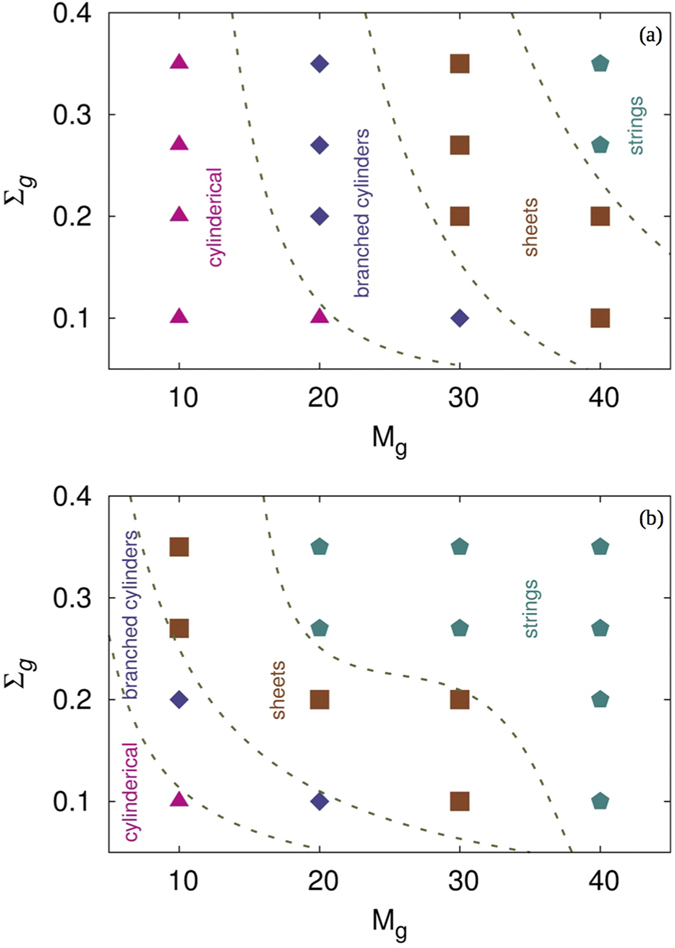
Self-assembly diagrams for systems with *W_SiNP_* =  a) 0.1 and b) 0.13. The lines that separate the different regions are merely guides to the eye.

For the lowest concentration *W*_*SiNP*_ = 0.07 with *M*_*g*_ = 10 and all grafting densities, the SiNP clusters (represented by grey spheres) formed are mostly spherical as expected (Fig. S2 of SI). As we increase the graft length, the clusters go from spherical to cylindrical and for higher graft densities and lengths, the clusters go back to forming multiple spherical aggregates. The self-assembly diagram for various graft lengths and densities with *W*_*SiNP*_ = 0.07 are shown in Fig. S4 of SI. Due to the high surface energy of SiNP, PCBM molecules (represented by transparent pink spheres in Fig. S2) migrate to the surface of the SiNP cluster and assume its shape. Hence, it is possible to control the shape of PCBM clusters by controlling the shape of SiNP aggregates. As the concentration of SiNP is very low, the clusters are small and do not span the simulation box, which is essential for a thorough connectivity of the acceptor material. As the concentration of SiNP increases in the system (*W*_*SiNP*_ = 0.1), we observe a larger variety of structures formed by SiNP and thus by PCBM as shown in the self-assembly diagram in Fig. 3(a). In these systems, for a graft length of *M*_*g*_ = 10 and all graft densities used in these simulations, the clusters are cylindrical. Similarly when *M*_*g*_ is increased to 20, for all the graft densities, branched structures of SiNP-PCBM clusters are formed. As *M*_*g*_ is increased to 30, a branched cylindrical structure of the SiNP-PCBM cluster is formed when the graft density is very low (Σ_*g*_ = 0.1). However, at this graft length, when the graft density is increased, a two dimensional sheet-like structures are formed. For systems with *M*_*g*_ = 40, two dimensional sheets are formed for lower graft densities (Σ_*g*_ = 0.1 and 0.2) while for higher graft densities (Σ_*g*_ = 0.27 and 0.35), string-like structures, that are highly percolated through all three dimensions of the box, are formed. This continuous network of PCBM cluster, formed with the aid of SiNP, is extremely useful for efficiently transferring electrons to cathode through the active layer of the solar cells.

To further analyze the effect of SiNP concentration on the morphology of PCBM domains, we increased the concentration of SiNP to 0.13 wt.%. We observe that, due to the increased number of SiNP, the formation of branched cylinders, sheets and strings occur at lower graft lengths than those in the *W*_*SiNP*_ = 0.1 case. Majority of the shapes are now sheets and strings with sheets forming at lower graft lengths and strings at higher *M*_*g*_ and Σ_*g*_ as shown in self-assembly diagram in Fig. 3(b). We also note that a cylindrical structure is formed only for *M*_*g*_ = 10 and Σ_*g*_ = 0.1. In the context of using these materials as the active layer in polymer-based solar cells, all these structures may prove useful for improving the exciton dissociation.

The mechanism of formation of anisotropic structures by P3HT-grafted SiNP is similar to that observed by Akcora *et al*.^66^ for systems with polystyrene (PS)-grafted silica nanoparticles. When two SiNPs come in contact with each other, the rearrangement of grafted chains make it more likely for the third particle to contact the pole diametrically opposite to the first contact. However, in this case since the grafted chains are stiff with a persistence length of ~37 Å, the relative loss in entropy due to the rearrangement of grafted chains is significantly lower than that for flexible polymers such as PS. Hence, the transition from one structure to the other occurs at a higher grafting parameters. Careful selection of graft densities and graft lengths is crucial for tuning the morphology of nanoparticles in the polymer. At low graft density and length, the enthalpic gain resulting from the core contacts dominates over the entropic loss arising from the rearrangement of grafted chains. Whereas, as the graft density and length increase, the entropic loss arising from the rearrangement of grafted chains to facilitate core contacts dominates over the enthalpic gain and hence under these conditions, it becomes thermodynamically favorable for the system to prevent internal rearrangement of nanoparticles resulting in more anisotropic structures, including branched cylinders, sheets, and interconnected strings. Also, at high graft densities, the local density of the grafted chains surrounding the nanoparticle clusters is significantly high that results in steric repulsions between these grafted chains and those that are tethered to nanoparticles approaching the cluster, thus preventing its growth in that direction.

**Figure 4:**
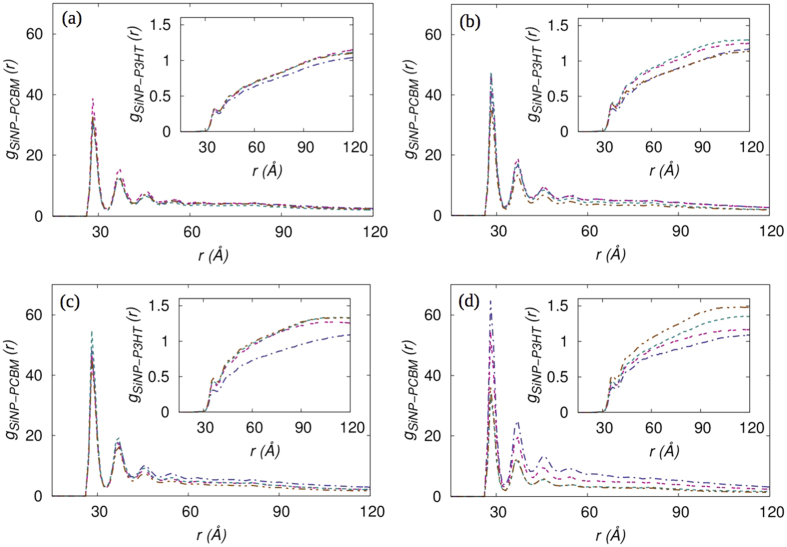
Partial pair correlation functions for SiNP-PCBM (Insets: SiNP-P3HT) with *W_SiNP_*  = 0.1 as function of r (\AA) for systems with Σ_*g*_ = a) 0.1, b) 0.2, c) 0.27, and d) 0.35. Dashed lines represent *Mg* = 10, double dashed lines represent *M_g_* = 20, dash dot dash represents *M_g_* = 30, and dash dot dot dash represents *M_g_* = 40.

The formation of connected network of structures by SiNP in turn improves the morphology of the PCBM clusters. The PCBM molecules are attracted towards the SiNP clusters due to the high surface energy of SiNP and the PCBM molecules easily spreads over the surface of the SiNP cluster. As the interaction parameter for SiNP-PCBM *χ*_*SiNP*−*PCBM*_ = −0.151 is higher than *χ*_*SiNP*−*P*3*HT*_ = −0.8913 and *χ*_*P*3*HT*−*PCBM*_ = −0.485, PCBM tends to more efficiently phase separate from P3HT and attach itself to SiNP. The tendency of PCBM to cover the SiNP surface more readily than P3HT prevents direct contact between SiNP and P3HT as shown in the partial pair correlation functions between SiNP-PCBM and SiNP-P3HT (inset) for systems with *W*_*SiNP*_ = 0.1 in Fig. 4. The results for *W*_*SiNP*_ = 0.07 and 0.13 are qualitatively similar and are shown in Figs S4 and S5 of the SI, respectively. The SiNP-PCBM partial pair correlation functions show the presence of multiple layers of PCBM around SiNP clusters for all *M*_*g*_ and Σ_*g*_, while the SiNP-P3HT functions indicate that the P3HT monomers are depleted from the surface of SiNP clusters. This is important with respect to the performance of this material as an active layer in polymer-based solar cells because, the dissociation of excitons occurs only at the donor (P3HT)-acceptor (PCBM) interface. As SiNPs are predominantly covered with PCBM and do have direct contact with P3HT, the presence of SiNP does not affect the exciton dissociation at the P3HT-PCBM interface.

### Effect of various structures on the performance of active layer

**Figure 5:**
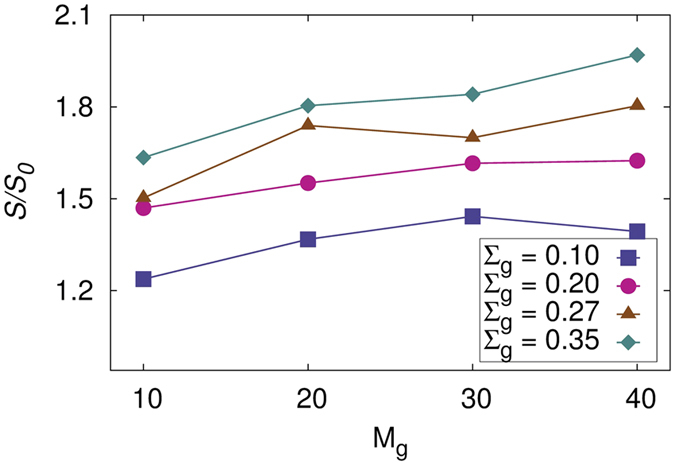
Normalized surface area of contact between P3HT and PCBM as a function of *M_g_* with *W_SiNP_ *= 0.1 for different grafting densities. Here, *S*_0_ is the surface area of contact for the system with no silica particles.

To verify whether the morphological changes brought about by the introduction of P3HT-grafted SiNP improve the efficiency of the active layer, we calculate several parameters that are known to significantly affect the device performance. A recent experimental work on pBTTT-PCBM blends have revealed that formation of star-shaped crystals improved the device performance^71^. An exciton that is formed within the polymer matrix upon absorption of light can successfully dissociate to give electrons and holes only at the donor-acceptor interface. This requires that the surface area of contact between P3HT and PCBM is optimum. A lower surface area of contact would result in lesser probability of dissociation and a very high surface area can only be possible when PCBM molecules are dispersed and thus do not contribute to the light-to-electricity conversion. Hence, it is required that the surface area of contact between the two materials be maximum while maintaining the interconnectivity of both domains within the system. We calculated the surface area of contact between P3HT and PCBM domains using the Voronoi tessellation of PCBM^72^. Only clusters of PCBM were considered for the calculation and isolated molecules were eliminated to avoid over estimation of the surface area. We first calculated the Voronoi faces of PCBM molecules and the surface area of contact between PCBM and P3HT was estimated as the sum of areas of all faces at the PCBM-P3HT interface. Figure 5 shows the normalized surface area of contact between P3HT and PCBM domains as a function of graft length and various graft densities for systems with *W*_*SiNP*_ = 0.1. Here, *S*_0_ is the surface area of contact between PCBM and P3HT in systems with no silica nanoparticles. The isolated PCBM molecules were again not considered for the calculation of *S*_0_ to maintain consistency. The results for systems with *W*_*SiNP*_ = 0.07 (Fig. S7) and 0.13 (Fig. S8) are qualitatively similar and are shown in the SI. We note that as both graft density and length increase, the surface area of contact between PCBM and P3HT also increases and for all the three concentrations of SiNP, at the highest graft density and length, the surface area becomes almost double that of the pure P3HT-PCBM system. This is because, as we increase the grafting parameters, the cluster shapes change from spherical to highly anisotropic networks that percolate through the simulations box. This drastically increases the surface area of PCBM that is in direct contact with the P3HT domains. We also note that for a given graft length and density, the surface area of contact is higher when the concentration of SiNP is higher.

**Figure 6:**
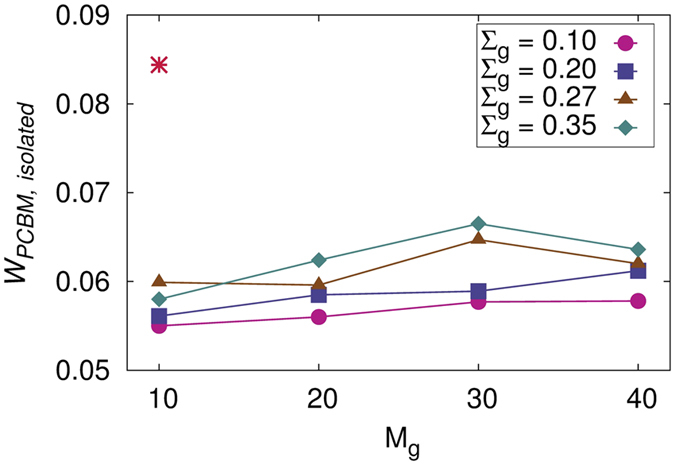
Weight fraction of isolated PCBM molecules as a function of *M_g_* for systems with *W_SiNP_* = 0.1 and different grafting densities. The weight fraction of isolated PCBM molecules in the system with no SiNP is represented by a^*^.

The next important factor in terms of improving the efficiency of polymer-based solar cells is the formation of excitons upon absorption of light by the polymer domains. It is known that the crystallinity of the polymer domain is crucial for the efficient formation and transport of excitons through the donor material. This is often hindered by the presence of dispersed PCBM molecules in the polymer domain. To quantify the effect of P3HT-grafted SiNPs on the purity of P3HT domains, we calculate the weight fraction of isolated PCBM molecules, employing the Voronoi tessellation method^24^, that do not completely phase separate during the annealing process. First the volume fraction of isolated PCBM molecules or small islands of PCBM clusters is calculated using the Voronoi volumes of all the cells of such particles as


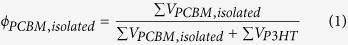


where, *V*_*PCBM*,*isolated*_ is the Voronoi volume of PCBM that are dispersed in the P3HT matrix and *V*_*P*3*HT*_ is the Voronoi volume of P3HT (matrix + graft) chains. The volume fraction *ϕ*_*PCBM*,*isolated*_ is then converted to weight fraction and plotted as a function of *M*_*g*_ for various Σ_*g*_ in Fig. 6 (c.f. Figs S8 and S9 for *W*_*SiNP*_ = 0.07 and 0.13, respectively). The weight fraction of isolated PCBM molecules in pure P3HT-PCBM system is also plotted for reference. For all the three concentrations of SiNP, we observe that the fraction of PCBM domains drops drastically upon addition of SiNP. We also note that as the graft length and density increase, the fraction of isolated PCBM molecules show a slight increase. This is intuitive because the SiNPs are grafted with P3HT chains and as the number of P3HT monomers around SiNP increases, they prevent the PCBM molecules from approaching closer to the surface of SiNP due to steric repulsions between the grafted P3HT monomers and the PCBM molecules. The fraction of isolated PCBM depends strongly on the concentration of SiNP. For the pure P3HT-PCBM system, the weight fraction of isolated PCBM is significantly higher than the systems with SiNP. This significant difference in the isolated PCBM molecules can lead to a rapid increase in the efficiency of BHJ due to the reduction in short circuit current. Due to the higher surface energy of SiNP and a more favorable interaction parameter *χ*_*SiNP*−*PCBM*_, introduction of SiNP to the P3HT-PCBM blend results in the migration of more PCBM to the SiNP surface. The weight fraction of isolated PCBM depends on the surface area available for PCBM-SiNP contact. As *M*_*g*_ and Σ_*g*_ are increased, the extent of reduction in the concentration of isolated PCBM is determined by two competing factors; One that favors migration of more PCBM to the SiNP surface due to the dramatic increase in the surface area due to the formation of branched structures and the other that prevents the PCBM molecules due to the steric repulsions between the grafted monomers and the PCBM molecules. Hence, we do not see significant differences in the fraction of isolated PCBM with increase in *M*_*g*_ and Σ_*g*_.

**Figure 7:**
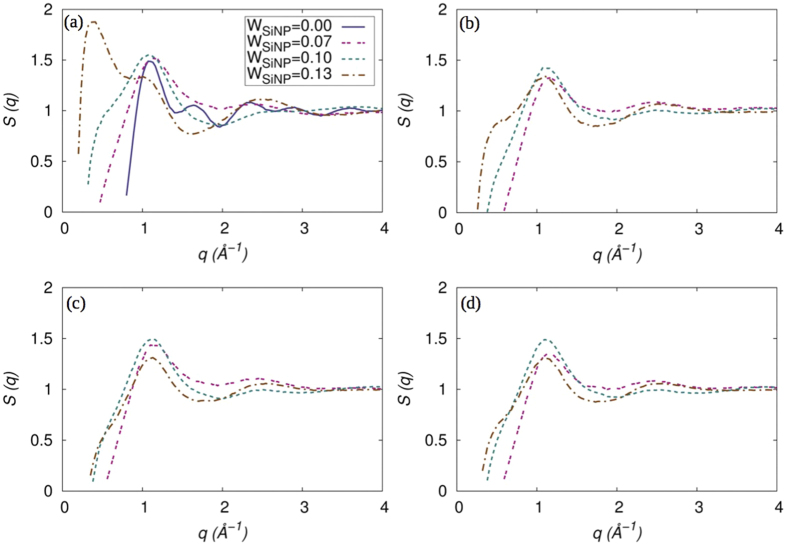
Static structure factor of P3HT at various SiNP loadings with *M_g_* = a) 10, b) 20, c) 30, and d) 40.

To study the effect of reduction in isolated PCBM on the crystallinity of P3HT domains, we calculate the static structure factor of P3HT in systems with *M*_*g*_ = 10, 20, 30, 40 and Σ_*g*_ = 0.1 for various concentrations of SiNP and compare the results with the system with no SiNP as shown in Fig. 7. We note that in all systems with varying concentrations of SiNP, a peak exists at *q* ~ 1.27 Å^−1^, which corresponds to the contact distance between two adjacent P3HT monomers. However, as the concentration of SiNP increases, we observe that a peak at *q* ~ 0.6 Å^−1^ develops and the intensity of this peak grows indicating a stronger correlation between P3HT units at longer distances. Reduction in the number of PCBM molecules that are isolated and dispersed within the P3HT matrix facilitates a tighter packing of the semiflexible P3HT chains resulting in enhanced crystallinity. However, as *M*_*g*_ increases, especially for cases with *W*_*SiNP*_ = 0.13, the PCBM clusters range from cylinders to branched cylinders to sheets to strings, the average P3HT domain size changes significantly. This appears to have a profound impact on the crystallinity of P3HT chains and is in excellent agreement with earlier studies^46^,^47^.

**Figure 8:**
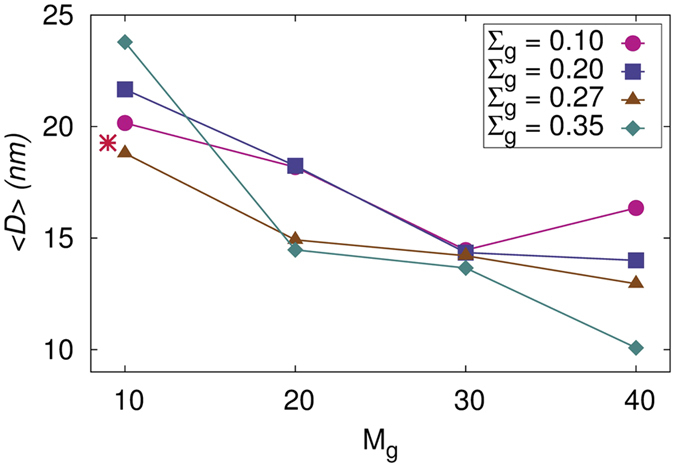
Average domain size of the P3HT phase as a function of *M_g_* for systems with *W_SiNP_* = 0.1 and different grafting densities.

Another important parameter that can dramatically alter the efficiency of these cells is the average domain size of the donor material. Based on the exciton diffusion length, it is imperative that the size of P3HT domains should be on the order of 10 nm such that an exciton formed within the polymer can encounter an interface before recombination. In our simulations, we calculated the average size of P3HT domains by first calculating the pore size distribution of the system with all P3HT chains removed. PCBM and SiNP clusters acts as pore cavity wall and the average size of the pore would then correspond to the average size of P3HT domains. PCBM beads that are dispersed in the matrix are removed while determining the pore size distribution to prevent underestimation of the domain size. The simulation box is first divided into grid points with a resolution of 0.25 Å and the location of each grid point is then optimized to maximize its distance from all the particles (PCBM and SiNP). If the distance of a grid point from surrounding beads is greater than radius of any of the surrounding bead, it indicates that the grid point is present in a space occupied by P3HT within the simulation box. The coordinates of such grid points are saved, together with the shortest distance. The saved data with the optimized locations of grid points along with the shortest distance allows determination of accessible positions for probes with different radii, with the shortest distance being the maximum radius of the probe molecule (*D*_*i*_/2). A cumulative histogram is then constructed and the pore size distribution *P* is equal to the negative derivative of Histogram with respect to *D*. The average domain size 〈*D*〉 is then calculated as


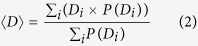


where, *D*_*i*_ is the size and *P*(*D*_*i*_) is its probability of occurrence. A detailed description of this method is provided elsewhere^73^. Figure 8 shows the average size of P3HT domains as a function of graft length for various graft densities. Our result for the system without SiNP agree very well with the experimental work of Wu *et al*.^74^ that estimated the average domain size of ~18 nm for thoroughly annealed samples. For all the three concentrations of SiNPs (Figs S10 and S11 of SI for *W*_*SiNP*_ = 0.07 and 0.13, respectively), we observe that the P3HT domain size decreases with increase in the graft parameters and for the highest graft length and density, the domain size approaches the ideal value suggested for optimum efficiency of the solar cell. This is because, for these grafting parameters, the PCBM cluster is percolated with highly interconnected strings.

## Conclusion

Using molecular dynamics simulations we study the effect of P3HT-grafted SiNP on the morphology of PCBM in P3HT for polymer-based solar cell applications. Addition of grafted SiNP drastically improves the morphology of P3HT-PCBM blends. Depending on the concentration and grafting parameters, SiNP self-assembles into a variety of structures ranging from cylindrical to branched cylinders to sheets to inter-connected strings. Due to the favorable interactions between SiNP and PCBM, PCBM migrates to the P3HT-SiNP interface and assumes the shape of SiNP within the system. This provides us the opportunity to indirectly control the spatial arrangement of PCBM without the requirement for any chemical modification of the species.

We also study the effect of SiNP on certain properties that are crucial for improving the efficiency of solar cells. We observe that by addition of SiNP, there is a significant improvement in the phase separation of P3HT-PCBM blends and also in the crystallinity of P3HT domains, which are crucial for improvement in exciton formation and dissociation. Due to the larger size and interaction parameters of SiNPs, they form the backbone structure for the PCBM domain and thus do not interfere with the exciton dissociation that occurs only at the P3HT-PCBM interface. Due to the network structure of PCBM domain, we find that it is possible to achieve a P3HT domain size of about 10 nm which has been proposed for maximizing the dissociation of excitons efficiently. Our results indicate that addition of grafted SiNPs to the P3HT-PCBM blends improves the morphology of the active layer that could in turn improve the overall light-to-electricity conversion in polymer-based solar cells.

## Additional Information

**How to cite this article**: Garg, M. and Padmanabhan, V. Addition of P3HT-grafted Silica nanoparticles improves bulk-heterojunction morphology in P3HT-PCBM blends. *Sci. Rep.* **6**, 33219; doi: 10.1038/srep33219 (2016).

## Supplementary Material

Supplementary Information

## Figures and Tables

**Table 1:** Bonded Interaction potentials for P3HT chains^21^.

Bond	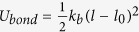	*k*_*b*_ = force constant, *l*_0_ = equilibrium bond length	*k*_*b*_ = 216.19 kcal mol^−1^ Å^−2^, *l*_0_ = 3.82 Å
Angle	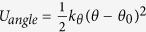	*k*_*θ*_ = force constant, *θ*_0_ = equilibrium bond angle	*k*_*θ*_ = 130.25 kcal mol^−1^ rad^−2^, *θ*_0_ = 2.65 rad
Dihedral	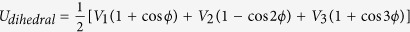	*V*_1_, *V*_2_, *V*_3_ are constants	*V*_1_ = 0.56 kcal mol^−2^,
			*V*_2_ = 1.08 kcal mol^−2^,
			*V*_3_ = 0.28 kcal mol^−2^

**Table 2:** Non-Bonded Interaction parameters for the P3HT-PCBM-SiNP system^21^,^59^.

Type 1	Type 2	*ϵ* (kcal mol^−1^)	*σ* (Å)
P3HT	P3HT	0.26	4.95
PCBM	PCBM	1.61	9.35
Si_6_O_12_	Si_6_O_12_	2.43	6.20
P3HT	PCBM	0.45	7.15
P3HT	Si_6_O_12_	0.47	5.58
PCBM	Si_6_O_12_	1.94	7.78
