# Supplementary material for: Addition of P3HT-grafted Silica nanoparticles improves bulk-heterojunction morphology in P3HT-PCBM blends
Source: Sci Rep. 2016 Sep 15;6:33219. doi: 10.1038/srep33219 (PMC5024111; doi:10.1038/srep33219)
Supplement: Supplementary Information [file srep33219-s1.pdf]

SUPPLEMENTARY INFORMATION

Addition of P3HT-grafted Silica nanoparticles improves bulk-heterojunction morphology in P3HT-PCBM blends

Mohit Garg and Venkat Padmanabhan*

Department of Chemical Engineering, Indian Institute of Technology (IIT), Kharagpur, West Bengal, 721302, India

*Corresponding author email: venkatp@che.iitkgp.ernet.in

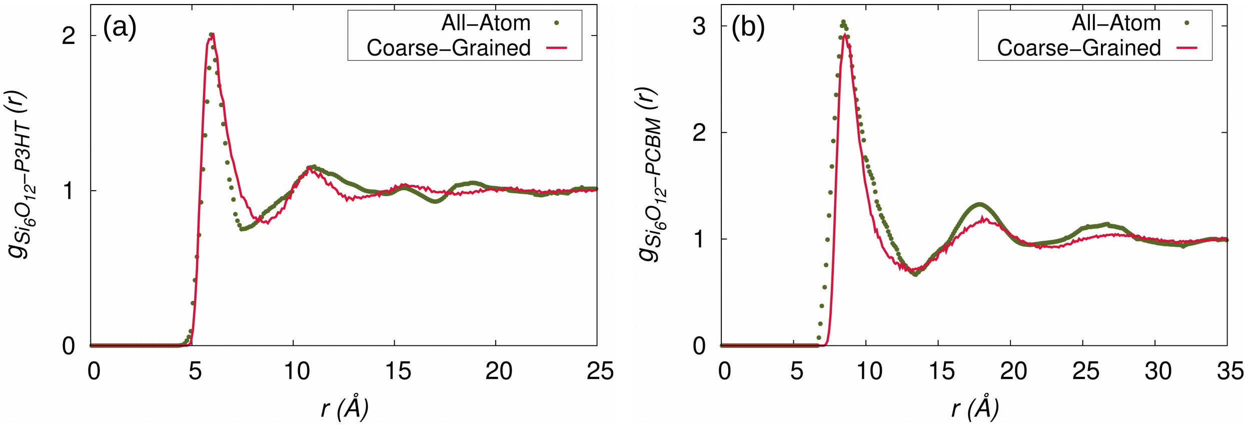

FIG. S 1. Partial pair correlation functions of a) Si_6O_{12}-P3HT and b) Si_6O_{12}-PCBM obtained from both coarse-grained and atomistic simulations.

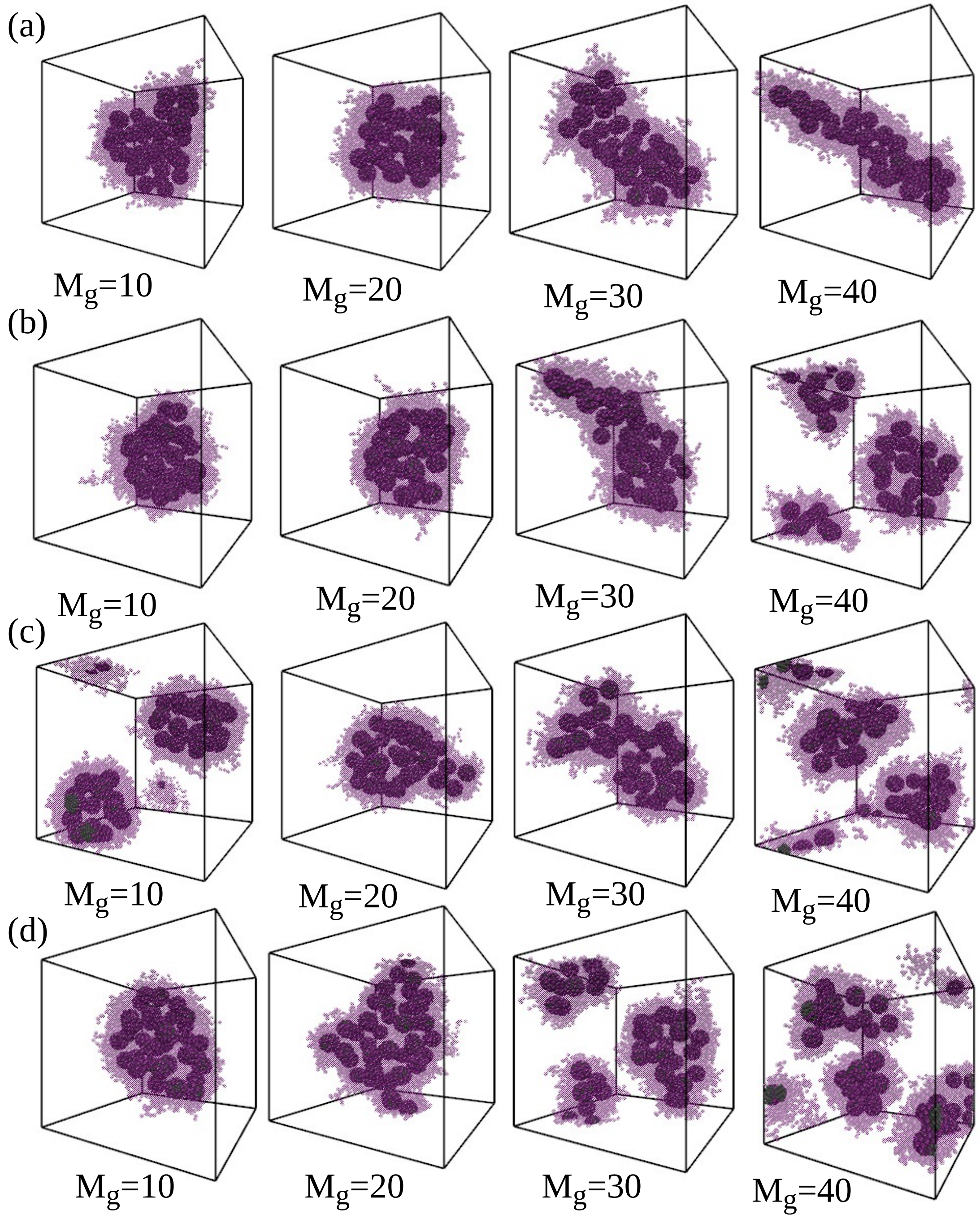

FIG. S 2. Equilibrated structures of SiNP and PCBM in P3HT. The transparent pink spheres represent PCBM molecules and the opaque grey particles represent SiNP in systems with $W_{SiNP} = 0.07$ and Σ_g = a) 0.1, b) 0.2, c) 0.27, and d) 0.35. The graft length (M_g) varies along the columns as indicated in the figure. The isolated PCBM molecules and P3HT are not shown for clarity.

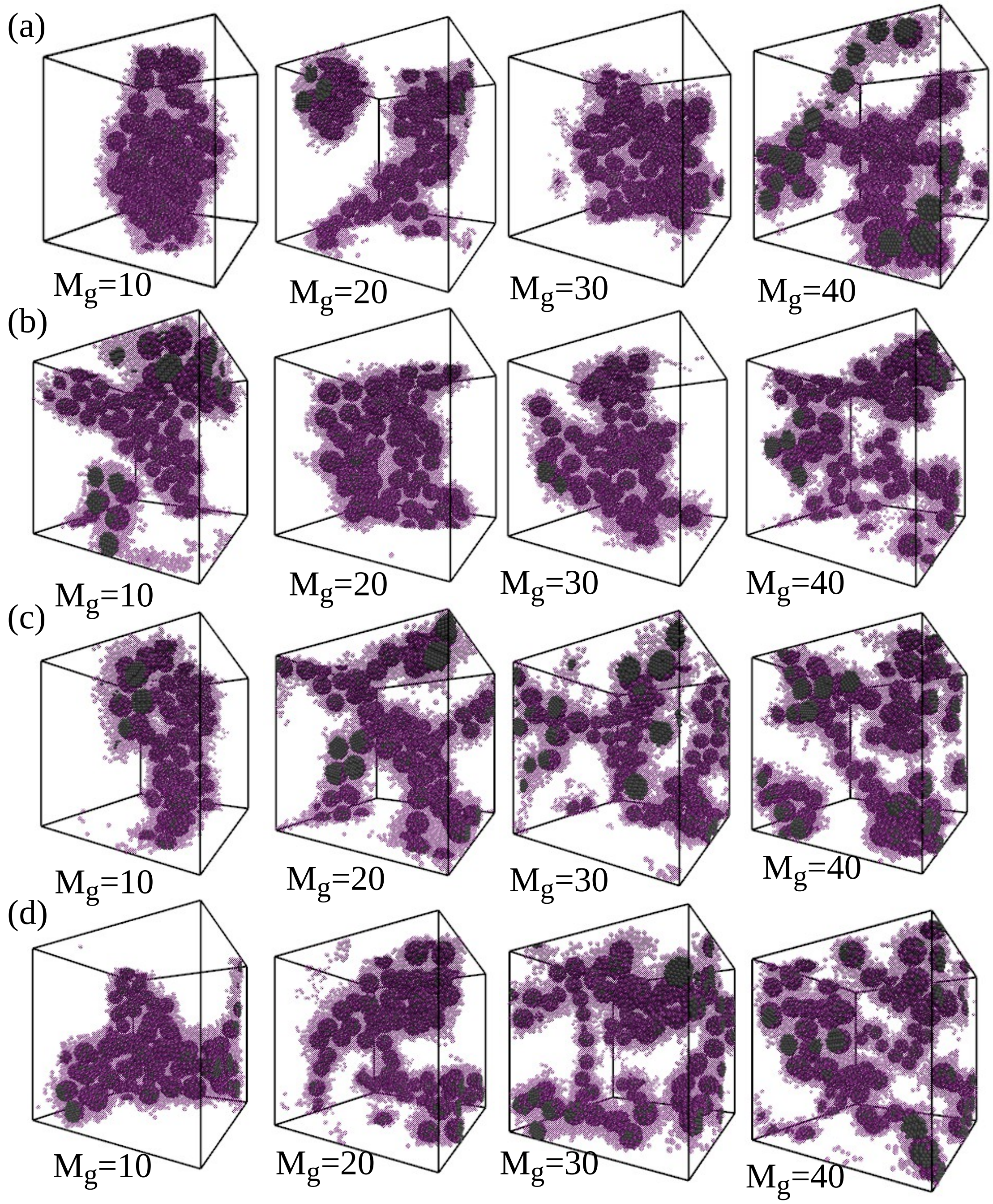

FIG. S 3. Equilibrated structures of SiNP and PCBM in P3HT. The transparent pink spheres represent PCBM molecules and the opaque grey particles represent SiNP in systems with $W_{SiNP} = 0.13$ and Σ_g = a) 0.1, b) 0.2, c) 0.27, and d) 0.35. The graft length (M_g) varies along the columns as indicated in the figure. The isolated PCBM molecules and P3HT are not shown for clarity.

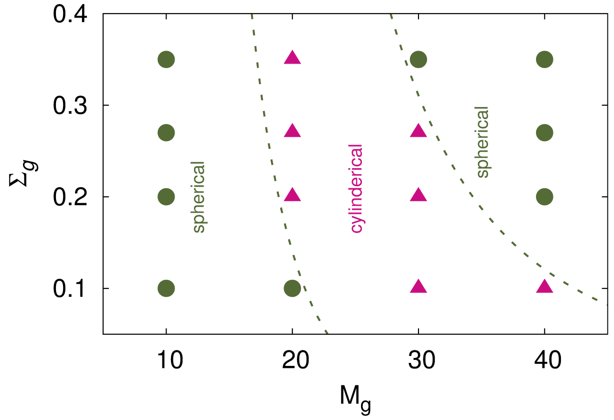

FIG. S 4. Self-assembly diagrams for systems with $W_{SiNP} = 0.07$. The lines that separate the different regions are merely guides to the eye.

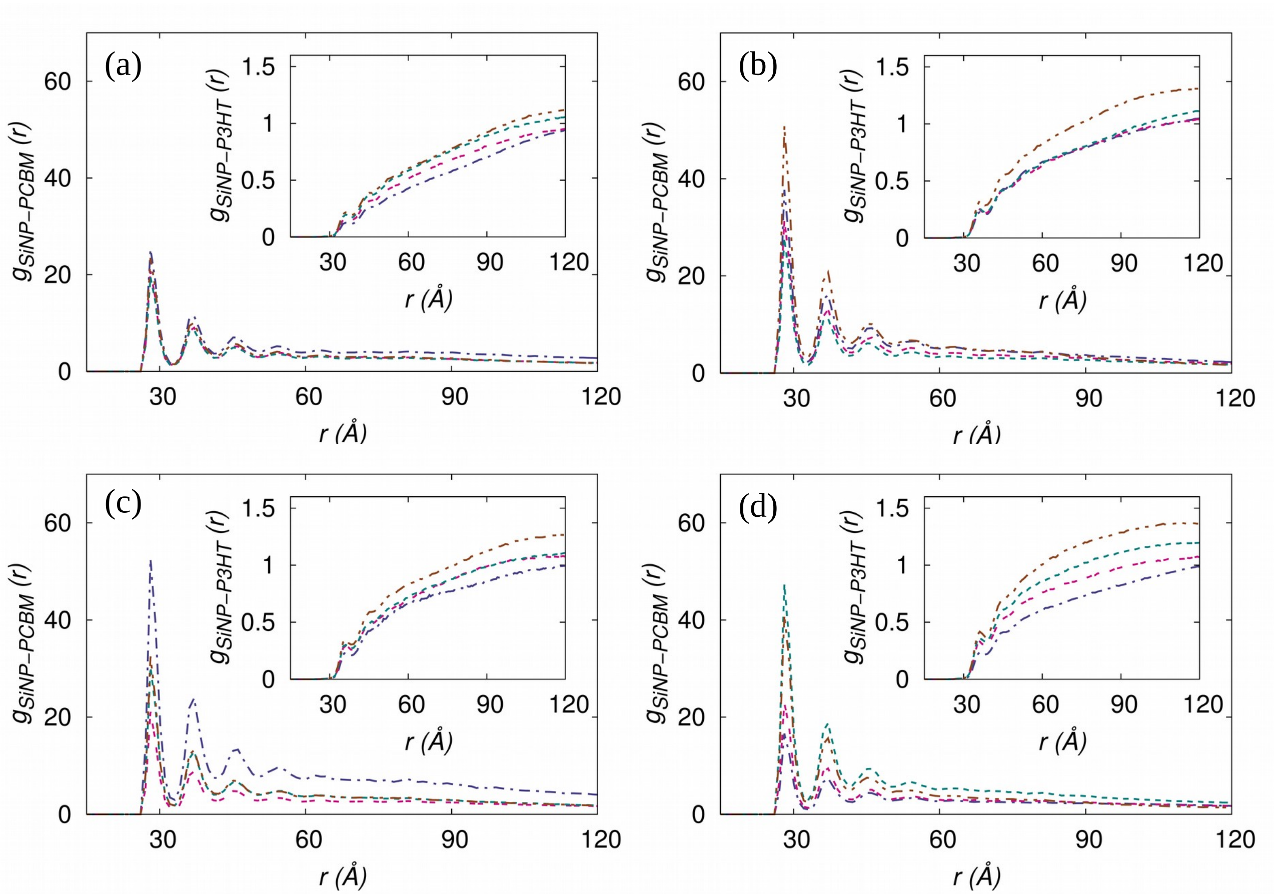

FIG. S 5. Partial pair correlation functions for SiNP-PCBM (Insets: SiNP-P3HT) with $W_{SiNP} = 0.07$ as function of r (Å) for systems with $\Sigma_g =$ a) 0.1, b) 0.2, c) 0.27, and d) 0.35. Dashed lines represent $M_g = 10$, double dashed lines represent $M_g = 20$, dash dot dash represent $M_g =$3 0, dash dot dot dash represent $M_g = 40$.

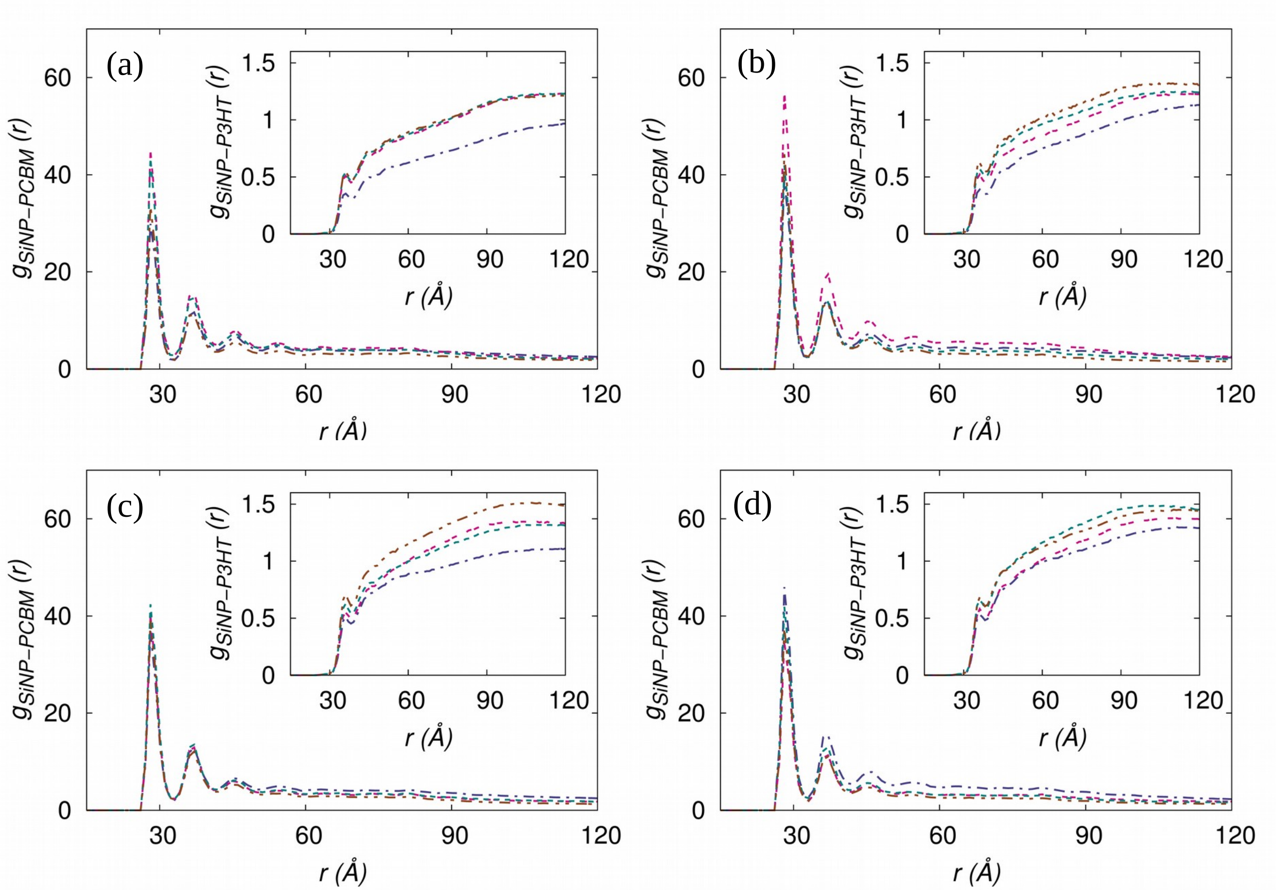

FIG. S 6. Partial pair correlation functions for SiNP-PCBM (Insets: SiNP-P3HT) with $W_{SiNP} = 0.13$ as function of r (Å) for systems with Σ_g = a) 0.1, b) 0.2, c) 0.27, and d) 0.35. Dashed lines represent $M_g = 10$, double dashed lines represent $M_g = 20$, dash dot dash represent M_g =3 0, dash dot dot dash represent $M_g = 40$.

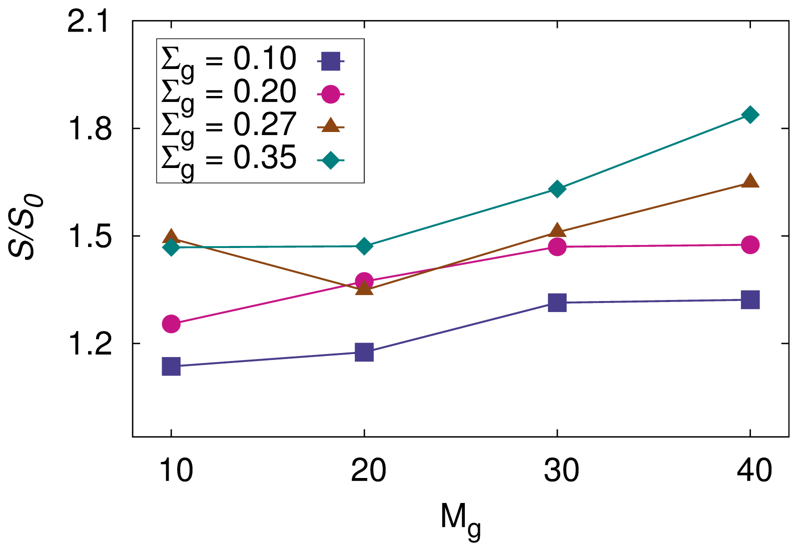

FIG. S 7. Normalized surface area of contact between P3HT and PCBM as a function of M_g with $W_{SiNP} = 0.07$ for different grafting densities. Here, S_0 is the surface area of contact for the system with no silica particles.

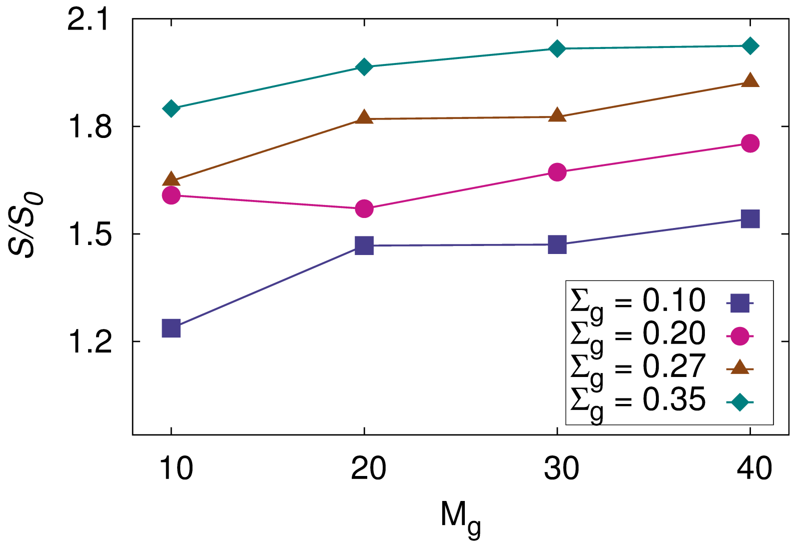

FIG. S 8. Normalized surface area of contact between P3HT and PCBM as a function of M_g with $W_{SiNP} = 0.13$ for different grafting densities. Here, S_0 is the surface area of contact for the system with no silica particles.

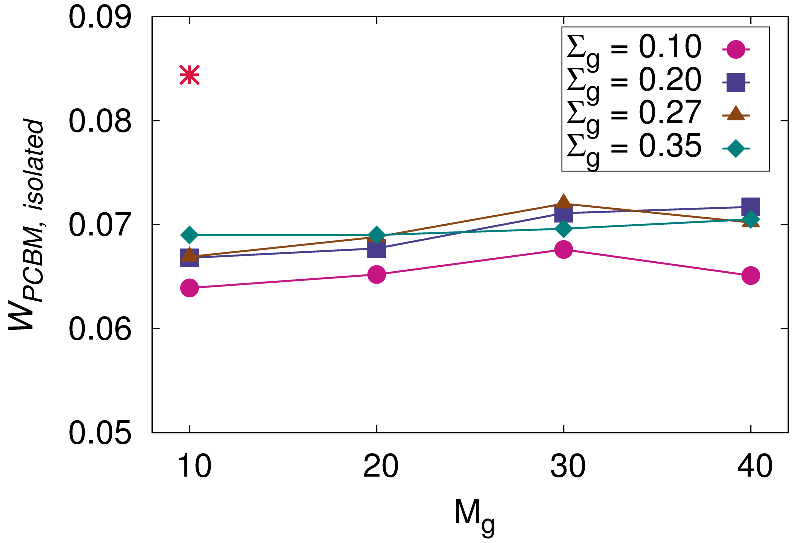

FIG. S 9. Weight fraction of isolated PCBM molecules as a function of M_g for systems with W_{SiNP} = 0.07 and different grafting densities. The weight fraction of isolated PCBM molecules in the system with no SiNP is represented by a *.

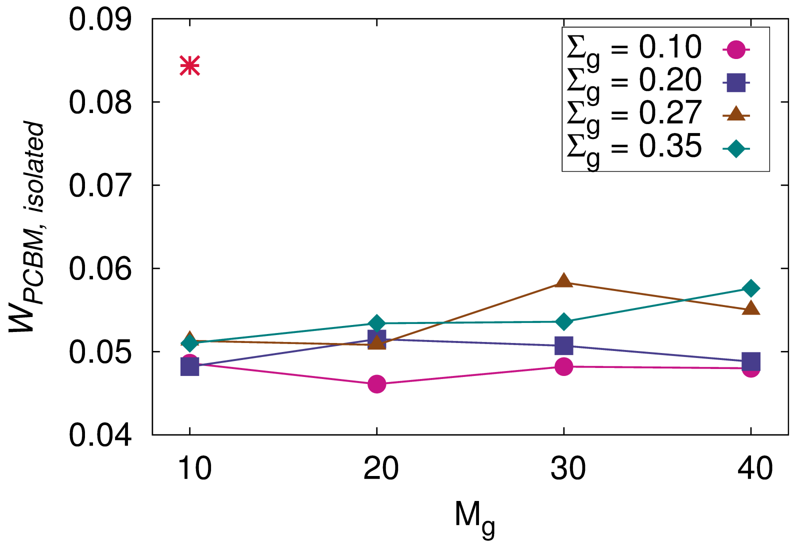

FIG. S 10. Weight fraction of isolated PCBM molecules as a function of M_g for systems with W_{SiNP} = 0.13 and different grafting densities. The weight fraction of isolated PCBM molecules in the system with no SiNP is represented by a *.

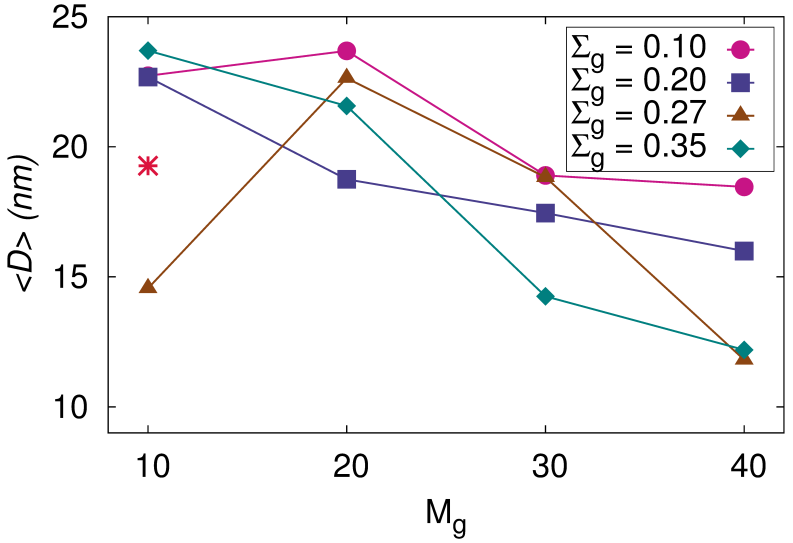

FIG. S 11. Average domain size of the P3HT phase as a function of M_g for systems with $W_{SiNP} = 0.07$ and different grafting densities.

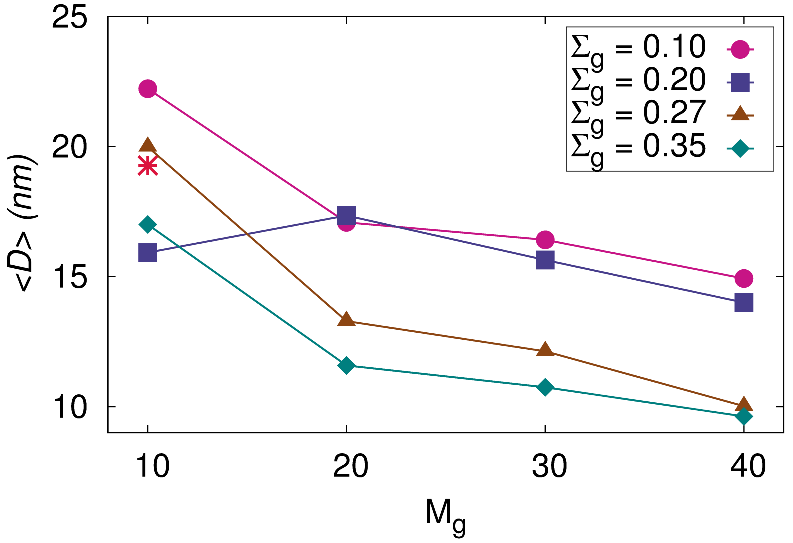

FIG. S 12. Average domain size of the P3HT phase as a function of M_g for systems with $W_{SiNP} = 0.13$ and different grafting densities.
